# Control of Blood Pressure and Risk Attenuation: Post Trial Follow-Up of Randomized Groups

**DOI:** 10.1371/journal.pone.0140550

**Published:** 2015-11-05

**Authors:** Tazeen H. Jafar, Imtiaz Jehan, Feng Liang, Sylvaine Barbier, Muhammad Islam, Rasool Bux, Aamir Hameed Khan, Nivedita Nadkarni, Neil Poulter, Nish Chaturvedi, Shah Ebrahim

**Affiliations:** 1 Program in Health Services & Systems Research, Duke-NUS Graduate Medical School, Singapore, Singapore; 2 Department of Community Health Science, Aga Khan University, Karachi, Pakistan; 3 Section of Nephrology, Department of Medicine, Aga Khan University, Karachi, Pakistan; 4 Centre for Quantitative Medicine, Office of Clinical Sciences, Duke-NUS Graduate Medical School, Singapore, Singapore; 5 Section of Cardiology, Department of Medicine, Aga Khan University, Karachi, Pakistan; 6 National Heart & Lung Institute, Imperial College, London, United Kingdom; 7 Institute of Cardiovascular Sciences, University College, London, United Kingdom; 8 Department of Non-communicable Disease Epidemiology, London School of Hygiene & Tropical Medicine, London, United Kingdom; University of Perugia, ITALY

## Abstract

**Background:**

Evidence on long term effectiveness of public health strategies for lowering blood pressure (BP) is scarce. In the Control of Blood Pressure and Risk Attenuation (COBRA) Trial, a 2 x 2 factorial, cluster randomized controlled trial, the combined home health education (HHE) and trained general practitioner (GP) intervention delivered over 2 years was more effective than no intervention (usual care) in lowering systolic BP among adults with hypertension in urban Pakistan. However, it was not clear whether the effect would be sustained after the cessation of intervention. We conducted 7 years follow-up inclusive of 5 years of post intervention period of COBRA trial participants to assess the effectiveness of the interventions on BP during extended follow-up.

**Methods:**

A total of 1341 individuals 40 years or older with hypertension (systolic BP 140 mm Hg or greater, diastolic BP 90 mm Hg or greater, or already receiving treatment) were followed by trained research staff masked to randomization status. BP was measured thrice with a calibrated automated device (Omron HEM-737 IntelliSense) in the sitting position after 5 minutes of rest. BP measurements were repeated after two weeks. Generalized estimating equations (GEE) were used to analyze the primary outcome of change in systolic BP from baseline to 7- year follow-up. The multivariable model was adjusted for clustering, age at baseline, sex, baseline systolic and diastolic BP, and presence of diabetes.

**Findings:**

After 7 years of follow-up, systolic BP levels among those randomised to combined HHE plus trained GP intervention were significantly lower (2.1 [4.1–0.1] mm Hg) compared to those randomised to usual care, (P = 0.04). Participants receiving the combined intervention compared to usual care had a greater reduction in LDL-cholesterol (2.7 [4.8 to 0.6] mg/dl.

**Conclusions:**

The benefit in systolic BP reduction observed in the original cohort assigned to the combined intervention was attenuated but still evident at 7- year follow-up. These findings highlight the potential for scaling-up simple strategies for cardiovascular risk reduction in low- and middle- income countries.

**Trial Registration:**

ClinicalTrials.gov NCT00327574

## Introduction

Hypertension is the leading attributable risk factor for death globally, including in South Asia where it accounts for a third of age-standardized deaths. [1] About 1 in 4 adults suffers from hypertension Pakistan. [2]

We previously reported 2-year outcomes of the Wellcome Trust funded Control of Blood Pressure and Risk Attenuation-(COBRA) trial (2004 to 2007) in Karachi, Pakistan. [3] The main trial was designed to assess the impact of family-based home health education (HHE) delivered every three months to households in randomized clusters, and a second approach of training general practitioners (GP) to manage hypertension optimally. We found that the combined strategy (HHE plus GP) was the most beneficial in lowering BP at 2 years, with a reduction of 5 mm Hg in systolic BP compared to no intervention. [3] However, the question remains as to whether such benefits are maintained, and translate to reductions in cardiovascular outcomes and mortality, which would increase the likelihood of wider adoption these interventions.

Few studies have examined the durability of public health interventions for hypertension. Long terms follow up of participants enrolled in efficacy trials of behavioral or drug interventions suggest benefit may persist and even translate into mortality reduction. For example, behavior counselling to lower dietary sodium has been shown to reduce cardiovascular morbidity in the long term. [4] The recent Action in Diabetes and Vascular Disease: Preterax and Diamicron Modified Release Controlled Evaluation (ADVANCE)-ON reported continued albeit attenuated benefit on mortality at 6 years post trial follow-up of BP lowering. [5] This benefit was attributed to BP differences observed during the in-trial period in ADVANCE. However, the long term effectiveness of public health interventions for BP lowering has not been evaluated in a low- and middle- income country (LMIC) setting where findings from high income countries may be less relevant.

We therefore conducted a 7- year follow-up of all the COBRA trial participants, inclusive of 2 year in-trial and 5- year post intervention period, to determine the sustained impact of the trial interventions on BP levels among adults in Karachi, Pakistan. We also tracked mortality and assessed other cardiovascular risk factors (tobacco use, weight, physical activity and lipids) among the trial participants.

We hypothesized that hypertensive adults randomized to HHE plus trained GP intervention will maintain lower BP than those randomised to the single or no intervention.

## Methods

Ethical approval was sought from the Ethics Review Committee at the Aga Khan University for the post intervention follow-up in 2011. Written informed consent was obtained from all participants.

### COBRA Trial Description

As previously described, the COBRA trial was a 2×2 factorial design cluster randomized controlled trial to determine the impact of family based HHE and/or special training of GPs on the BP levels of adults with hypertension (registration number NCT00327574, ClinicalTrials.gov). The sampling frame and study design details have been described previously. [3] Briefly, 12 communities (with about 250 households each) were randomly selected from middle to low income areas in Karachi using multistage cluster sampling techniques. Individuals aged 40 years or older with known hypertension or with consistently elevated BP on two separate visits (mean of last two of three measurements of systolic pressure ≥140 mm Hg or mean diastolic pressure ≥90 mm Hg) and residing in the 12 clusters were eligible for inclusion in the study. The 12 clusters were randomly assigned to four groups of 3 clusters each: HHE, trained GP, HHE plus trained GP, and no intervention, using computer generated codes by the data manger. A total of 1341 eligible adults resident in the 12 clusters were included in the trial. [3]

#### Home Health Education

Trained community health workers (CHWs) visited households to advise participants how to eat healthily (reduce sodium, total and saturated fat consumption, increase fruit, vegetable and low fat dairy product intake), stop smoking, maintain a normal body weight, and increase physical activity. In addition, they emphasized the importance of adherence to BP medication and physician follow up. Home visits were made every quarter for the duration of the trial.

#### General Practitioner Education

All GPs in the six study areas assigned to this intervention were invited for training, with the realistic aim to train at least two-thirds of all GPs from each area. The training was a one day session focused on standard treatment for the management of hypertension, based on the seventh report of the Joint National Committee (JNC-7) and the Fourth Working Party of the British Hypertension Society guidelines modified for the Indo-Asian population.[6,7] All study participants aged 40 years or above with hypertension were advised to consult a local GP. Those in the clusters randomized to the trained GP arm were given a list of trained GPs within their cluster.

#### Screening and Recruitment

All households in each cluster were visited by trained research staff masked to randomization status, and informed consent was obtained for screening from all adults aged 40 and above, who then underwent 3 BP measurements with a calibrated automated device (Omron HEM-737 IntelliSense; Omron Healthcare Inc., Vernon Hills, Illinois, US) in the sitting position after 5 minutes of rest. [8]Those with an elevated BP and not on antihypertensive medication were visited again for re-measurement of BP 1–4 weeks after the initial visit. If the mean BP remained elevated, these individuals were invited to participate. In addition, those with known or treated hypertension were also invited to participate, irrespective of measured BP.

#### In-Trial Follow-up during 2 years of Intervention

Hypertensive adults were evaluated by trained field staff in all 4 groups, masked to randomization status. Three measurements of BP were taken at 4-monthly intervals during 2 years.

### Post Intervention Follow-up

In 2012, seven years post randomization inclusive of two years of in-trial follow-up visits, trained outcomes assessors masked to randomization status visited the homes of all participants to establish contact and track vital status. Individuals were invited to participate in the post-trial follow-up study and written informed consent was obtained. A standardized questionnaire on diet, physical activity, antihypertensive medications, and hospitalizations was administered by trained interviewers.

BP was measured with the same calibrated automated device and using the same techniques as in the main COBRA trial, and BP measurements were repeated after two weeks (i.e. 2 sets of readings 2 weeks apart).

The frequency and interval of the visits were balanced across the four randomized groups and planned during the first half of the day to minimize seasonal and diurnal variation in measurements, respectively. [9] Repeat visits were arranged for those not present at a suitable date.

Fasting blood was collected on the participants and lipid profile was measured (HITACHI-912, Roche Japan) at the Aga Khan University laboratory certified by the College of American Pathologists.

A dedicated field team was employed to track all subjects lost to follow-up and those who had relocated to other clusters within the city of Karachi. For individuals who had reportedly died, the next of kin or nearest relative was contacted for information on the cause and date of death and efforts were made to review the death certificate (from the hospital and/or the graveyard) and track the district mortality register.

### Quality Assurance

A randomly selected 10% of participating households were contacted by telephone or revisited by an independent team for validation of key information. In addition, all households with reported hospitalizations and deaths were re-contacted for tracking details.

### Analysis

#### Primary Outcomes

The two primary outcomes were:

1. change in systolic BP from baseline to post-trial follow-up visit; and
2. all-cause mortality.

#### Secondary Outcomes

The change from baseline to post trial follow-up in the following: diastolic BP, proportion with controlled BP, body mass index (BMI), waist hip ratio, percent current tobacco users, total physical activity, and LDL-cholesterol. Change in self-reported adherence to anti-hypertensive medications was also evaluated.

#### Statistical Analysis

We used STATA version 12 for statistical analyses. All main analyses were on an intention-to-treat (ITT) principle. We considered a 2-sided P value less than 0.05 to be statistically significant for the main effects and a value less than 0.1 to be significant for interactions. We report adjusted differences in means and 95% CIs for the treatment effects, compared to no intervention (usual care). As in the 2- year follow-up analyses, all 7 -year analyses accounted for clustering by household at the census level as a random effect in the main analysis; this was also the unit of randomization.

The multivariable model was adjusted for clustering, age at baseline, sex, baseline systolic and diastolic BP, and presence of diabetes. Generalized estimating equations (GEE) were used to analyze the primary and the secondary outcomes (BP-related outcomes, and behavioral and clinical outcomes). Since the focus of this work is on the population averaged parameter and the intra class correlation (ICC) within clusters was negligible, the GEE was employed for the purpose of analysis adjusting for clustering as robust standard error at the household level as the unit of analysis. [10] The GEE approach allows all non-missing data to be used in the analysis without imputation. For example, BP measurement obtained during 2 years of follow-up were used for individuals with missing BP readings at 7 years [11,12] The underlying assumption for the purpose of this analysis is that data are missing at random. All multivariable models were also adjusted for age, sex, baseline value of the outcome, and, diabetes when relevant. Since interaction between HHE and trained GP intervention had been shown in the analysis of the previous time-point, it was also tested.

The log-rank test was used to characterize the difference in survival among the randomized groups. Cox proportional hazards frailty models were used accounting for clustering and other covariates of interest to estimate the hazard ratio for time to death.

In the analysis of the secondary outcomes, we compared the proportion of participants with controlled BP at the last visit among randomized groups by using a GEE as elaborated above, accounting for clustering of households at the census level, age, sex, and baseline BP control and diabetes using the following definitions of BP control 1) systolic BP <140 mm Hg and diastolic BP <90 mm Hg; 2) systolic BP <140 mm Hg; and 3) systolic BP <160 mm Hg.

We performed additional comparative analyses among randomized groups using GEE to determine whether lifestyle and treatment targets advised during intervention were achieved or sustained during post trial follow-up.

#### Sensitivity Analysis

We also performed sensitivity analyses on our findings after 1) excluding deaths; 2) ITT after adding 1 mm Hg systolic BP to all imputed values for individuals with missing BP at 7 year follow-up; 3) ITT after adding 2 mm Hg systolic BP to all imputed values for individuals with missing BP at 7 year follow-up; and 4) ITT after carrying the last available reading forward for those who were dead and replacing missing readings of those lost to follow-up with the mean follow-up BP in each of the 12 clusters; 5) ITT after replacing all missing readings with the mean follow-up BP in each of the 12 clusters; 6) ITT after multiple imputation, in which missing values of 7 year follow-up systolic BP (52.4%) were replaced through a process of multiple imputation using SAS 9.3. Variables were included in the imputation model if they were risk factors for hypertension, or were correlated with systolic BP at year 7 or its missingness. Therefore, the imputation model included baseline age, gender, education, intervention group, BMI, smoking, physical activities score, antihypertensive medication use, history of diabetes, and systolic BP at baseline, as well as all available systolic BP during the 2 years of in-trial follow-up. The resultant dataset allowed us to use data from all 1341 individuals randomized. Twenty multiply-imputed datasets were created using fully conditional specification (FCS) predictive mean matching methods under the missing at random assumptions. [13,14] After imputation, each of the 20 datasets was analyzed using a GEE model similar to the approach used for the non-imputed dataset. The parameter estimates obtained from each dataset were pooled for inference.

In addition, the main analysis was repeated after subdividing participants into those with and without diabetes. The use of self-reported anti-hypertensive medications was compared among individuals who responded during the final follow-up (n = 740).

## Results

**Fig 1:**
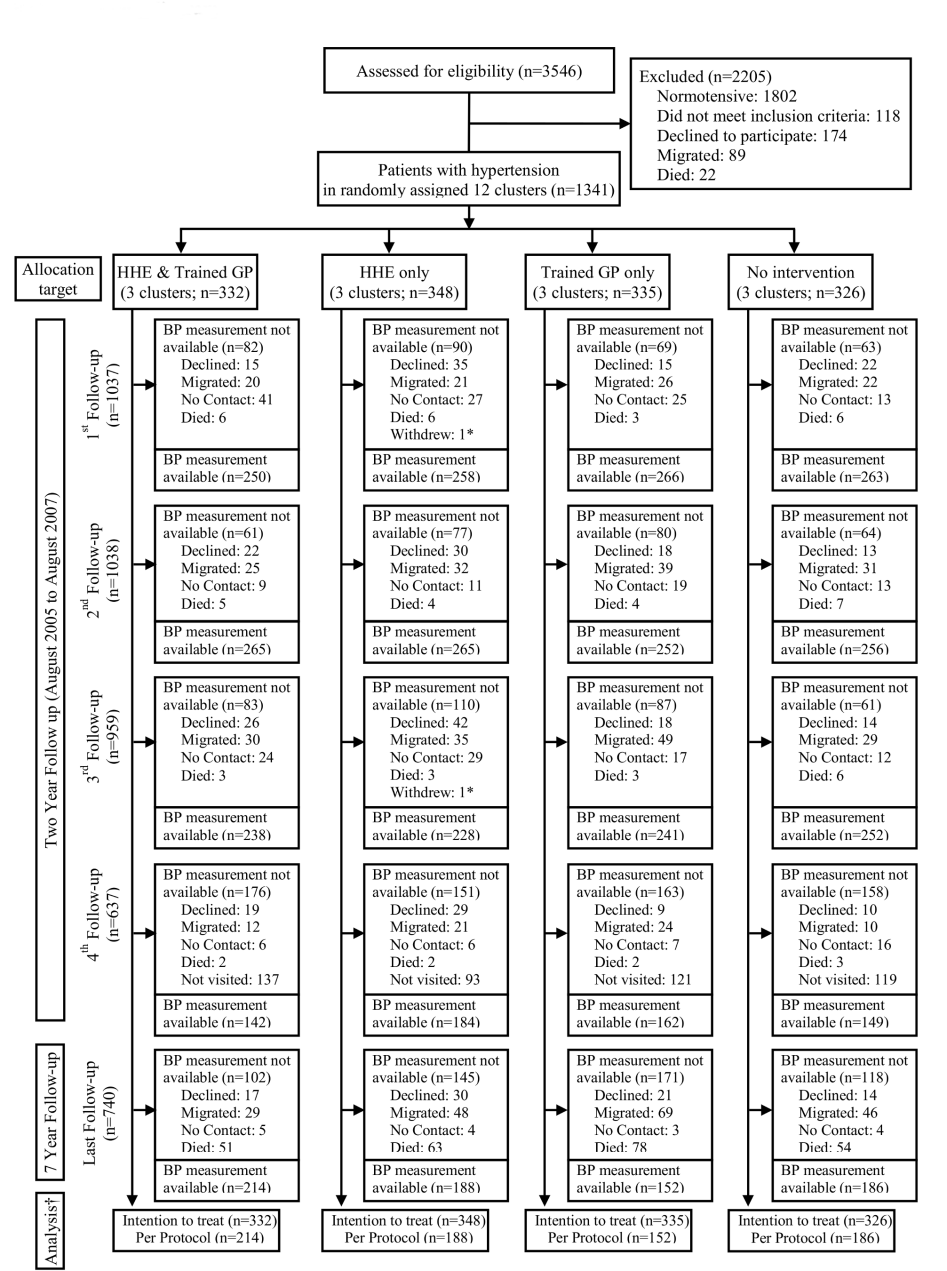
Study flow diagram. HHE = Home Health Education; GP = General Practitioner. * One pregnant woman & one man withdrew because of target organ damage & severe debility. † Participants who successfully completed 7 years of follow-up were included in the per protocol analysis. Note: The status of excluded participants at each follow-up is variable except deaths.

The consort diagram is shown in Fig 1. The baseline characteristics of the clusters and participants are shown in Table 1. The baseline characteristics of those alive at post trial visit are shown in Table A in S1 File (Socio-demographic characteristics of Responders versus Non-responders for Blood Pressure level at post trial follow-up by Randomized Groups).

**Table 1:** Comparison of Clusters and Baseline Participant Characteristics among Randomized groups.

Characteristics	GP + HHE*	HHE only	GP only	Usual Care
**Cluster Characteristics**				
**Number of clusters**	3	3	3	3
**Number of Households**	656	673	657	664
**Average number of residents per household**	6.5 (3.0)	6.7 (3.0)	6.6 (3.0)	6.4 (3.1)
**Participant Characteristics**				
**Number of patients with hypertension**	332	348	335	326
**Age in years mean (SD)**	54.0 (11.5)	52.7 (11.4)	55.3 (11.5)	53.3 (11.5)
**Body mass index**	27.6 (5.2)	25.9 (5.0)	26.2 (5.6)	27.0 (5.6)
**Men n (%)**	112 (33.7)	133 (38.2)	138 (41.2)	118 (36.2)
**Education Level n (%)Illiterate**	131 (39.5)	110 (32.8)	151 (43.4)	162 (49.7)
**Primary or higher**	201 (60.5)	197 (56.6)	225 (67.1)	164 (50.3)
**†** **Tobacco n (%)PastCurrentNever**	37 (11.1)101 (30.4)194 (58.4)	33 (9.5)142 (40.8)173 (49.7)	49 (14.6)110 (32.8)176 (52.5)	25 (7.7)110 (33.7)191 (58.6)
**‡** **Physical activity MET score ≥ 840 n (%)**	96 (28.9)	116 (33.3)	96 (28.7)	148 (45.4)
**§** **Low SES n (%)**	232 (69.9)	246 (70.7)	188 (56.1)	266 (81.6)
|| **Diabetes n (%)**	76 (24.0)	95 (28.8)	105 (33.0)	94 (29.9)
**Waist circumference Mean (SD) cms**	93.3 (12.2)	90.9 (11.9)	91.1 (11.4)	90.8 (12.2)
¶ **Central Obesity n (%)**	269 (81.0)	254 (73.0)	244 (72.8)	241 (73.9)
**††** **Overweight/Obesity n (%)**	274 (82.5)	250 (72.0)	236 (70.7)	252 (77.8)
**Systolic BP Mean (SD) mm Hg**	148.3 (24.7)	151.8 (24.5)	153.3 (24.62)	153.3 (24.6)
**Diastolic BP Mean (SD) mm Hg**	91.1 (13.0)	93.7 (12.9)	92.9 (12.9)	95.5 (12.5)
**LDL-cholesterol mg/dl**	121.2 (33.2)	117.1 (30.8)	117.6 (34.6)	122.2 (31.5)
**‡‡** **Controlled BP n (%)**	94 (28.3)	57 (16.4)	93 (27.8)	70 (21.5)
**Receiving anti-hypertensive Medication n (%)**	117 (35.2)	138 (39.7)	119 (35.5)	132 (40.5)
**§§** **CVD Risk score Mean (SD)**	0.21 (0.18)	0.23 (0.19)	0.25 (0.19)	0.24 (0.19)

* GP = trained general practitioner; HHE = home health education

† Use of Tobacco (Smoking and Chew). Current users are those who at the time of survey either smoke and/or use pan with tobacco. Past users are those who have smoked **≥** 100 cigarettes or chewed **≥** 100 pans in their life time. Never users are those who have smoked **<**100 cigarettes or chewed **<** 100 pans in their life time.

‡ Physical activity MET (Metabolic Equivalent) Score was defined as: Total MET-minutes/week = Walk (METs-min*days) + Moderate (METs*min*days) + Vigorous (METs*min*days).

§ Low socioeconomic status defined as monthly household income <$50 as reported by Federal Bureau of Statistics.

|| Diabetes was defined as patients taking anti diabetic medications, or having fasting blood sugar ≥ 7.0 mmol/L (126 mg/dL). Blood samples were missing in 62 subjects.

¶ Central obesity was defined as having a waist circumference of ≥ 80 cm in women and ≥ 90 cm in men.

†† Overweight/Obesity was defined as Asian specific criterion of ≥23 kg/m^2^.

‡‡ Controlled BP is defined as (systolic BP < 140 & diastolic BP < 90 mm Hg).

**§§** CVD risk score is computed based on Framingham risk equation based on age, total cholesterol, HDL-cholesterol, systolic BP, antihypertensive therapy status, smoking, and diabetes.

### Response Rate During Post Trial Follow-up

At 7- year follow-up, 311 (23.2%) of the original cohort had reportedly died. Comparison of baseline characteristics between those alive (n = 1030) vs dead (n = 311) suggests a healthy survivor bias with those alive more likely to be younger, with lower systolic BP, and less diabetes at baseline across all randomized groups. (Table A in S1 File). BP measurements were available on 740 (72%) of the remaining cohort targeted for recruitment at follow-up (n = 1030). However, comparison of characteristics between those alive at post trial follow-up with (n = 740) and without BP measurement (n = 290) did not reveal significant differences in baseline characteristics. (Table A in S1 File: Socio-demographic characteristics of Responders versus Non-responders for Blood Pressure level at post trial follow-up by Randomized Groups).

Quality assurance checks on key measurements (follow-up visit, standardization method, deaths and no contacts) revealed <1% inconsistency.

### Change in Blood Pressure

By the end of the 7 year follow up (inclusive of 5 years post-intervention, systolic BP decreased in all 4 groups (Table 2). However, compared to the no intervention group there was a significantly greater decline in the adjusted difference in systolic BP of 2.1 mm Hg in the combined HHE plus GP group (p = 0.04), and of 1.2 mm Hg in the HHE -only groups (p = 0.02) (Table 2).

**Table 2:** Outcomes- Blood Pressure Levels and control rates among Randomized Groups at 7 Years.

	HHE + Trained GP β (95% CI β) (n = 317)	HHE only β (95% CI β) (n = 330)	Trained GP only β (95% CI β) (n = 318)	Usual Care β (95% CI β) (n = 314)
**†** **Mean systolic BP mm Hg (95% CI)**	146.6 (144.2, 149.0)	149.0 (145.1, 152.9)	150.1 (143.1, 157.1)	151.9 (150.6, 153.1)
^**a**^ **Mean change in systolic BP (95% CI)**	-2.1 (-4.0, -0.1)	-1.2 (-2.2, -0.2)	-1.2 (-2.6, 0.2)	reference
**p-value**	0.040	0.022	0.090	
**†** **Mean diastolic BP mm Hg (95% CI)**	86.5 (82.7, 90.4)	86.9 (83.1, 90.6)	86.6 (81.8, 91.4)	87.6 (85.3, 89.9)
^**a**^ **Mean change in diastolic BP (95% CI)**	-0.1 (-1.4, 1.3)	-0.1 (-1.4, 1.3)	-0.1 (-1.5, 1.2)	reference
**p-value**	0.889	0.919	0.855	
**Blood pressure control: risk ratio (systolic BP<140 And diastolic BP <90) (95% CI)** ^**a**^	1.09 (0.86, 1.38)	1.17 (0.99, 1.39)	0.91 (0.75, 1.11)	reference
**p-value**	0.454	0.064	0.366	
**Blood pressure control** ^**1**^ **: risk ratio (systolic BP<140)(95% CI)** ^**a**^	1.01 (0.80, 1.29)	0.92 (0.78, 1.07)	0.80 (0.61, 1.05)	reference
**p-value**	0.912	0.287	0.108	
**Blood pressure control** ^**2**^ **: risk ratio (systolic BP<160)(95% CI)** ^**a**^	1.15 (1.01, 1.30)	1.14 (1.03, 1.26)	1.10 (0.94, 1.29)	reference
**p-value**	0.032	0.013	0.218	

GP = general practitioner; HHE = home health education.

**†** Mean follow-up systolic and diastolic BP levels are based on individuals with available BP readings at 7 years post trial follow-up (n = 740)

^a^ Change is the difference between baseline minus follow-up with negative sign indicating a decline.

Adjusted for clustering, time, age, gender, diabetes status, and baseline systolic blood pressure, diastolic blood pressure, or blood pressure control, respectively. P values indicate comparison with usual care (reference). Adjusted risk ratios computed from adjusted marginal probabilities using logistic generalized estimating equations model (Santos, Carlos AST, et al. "Estimating adjusted prevalence ratio in clustered cross-sectional epidemiological data." BMC medical research methodology 8.1 (2008): 80).

Age and baseline systolic BP were significantly associated with change in BP in the multivariable model. The previously noted interactions between treatment groups HHE and trained GP on change in systolic BP at 2 years were not apparent at 7 years (p = 0.78). The p value for interaction was 0.08 for the outcome of controlled BP (<140/90mm Hg). The intraclass cluster coefficient (95% CI) for change in SBP was 0.0009 (<0.0001 to 0.0116).

There was no difference in controlled BP using the conventional definition of <140/90 mm Hg among randomized participants. However, severely uncontrolled systolic BP (systolic BP 160 mm Hg or more) was less common in the combined HHE plus GP group than in the usual care group (p = 0.03) (Table 2).

Diastolic BP also declined in all 4 groups. However, there was no significant difference in decline in diastolic BP among randomized groups.

### Mortality

A total of 311 (23.1%) participants died during 7 years of follow-up. 55% of these deaths were at home; 40% in the hospital, and 5% on the way to the hospital. A copy of death certificate (any source) was available for 46% (n = 144) of all reported deaths. As shown in Table 3, although mortality was lower in the combined intervention group compared to control, with a hazard ratio (95% CI) of 0.81 (0.44 to 1.46), the confidence intervals were wide in both unadjusted and adjusted models. Moreover, the test for proportional hazard assumption yielded p = 0.05 suggested marginal performance of the Cox model. 45% of all deaths were reportedly attributed to CVD by next-of-kin, and this proportion was not different among the randomized groups.

**Table 3:** Outcomes- Hazard Ratio for All-cause mortality Among Randomized Groups.

Treatment Group	Number Deaths	*Unadjusted Hazard Ratio (95% CI) All-cause Mortality	**Adjusted Hazard Ratio (95% CI) All-cause Mortality
**HHE & Trained GP (N = 332)**	67	0.82 (0.47 to 1.42)	0.81 (0.44 to 1.46)
**HHE only (N = 348)**	78	0.95 (0.77 to 1.17)	1.00 (0.80 to 1.26)
**Trained GP only (N = 335)**	90	1.25 (0.90 to 1.73)	1.00 (0.73 to 1.36)
**Usual Care (N = 326)**	76	reference	reference

HHE = home health education; GP = general practitioner.

* Unadjusted analysis accounts for clustering

** Adjusted for clustering, age, gender, baseline systolic BP, baseline diastolic BP, diabetes, and smoking

### Life style and LDL-cholesterol measurements

The adjusted differences in randomized groups compared to usual care in changes in BMI, waist circumference, tobacco use, physical activity, and LDL-cholesterol levels from baseline to follow-up are shown in Table 4. While no significant differences were detected among randomized groups for most parameters, the adjusted difference in LDL- cholesterol was a greater decline by 2.7 (4.8 to 0.6) mg/dl in the combined HHE plus trained GP group compared with those allocated to no intervention. (p = 0.01).

**Table 4:** Change in Behavioral and Clinical outcomes at 7-year follow-up.

	*HHE and GP groupβ (95% CI β)	HHE only groupβ (95% CI β)	GP only groupβ (95% CI β)	Usual Careβ (95% CI β)
**†** **Mean BMI kg/m2 (95% CI)**	28.2 (27.3, 29.0)	27.5 (26.9, 28.1)	26.6 (25.8, 27.4)	27.1 (25.6, 28.6)
^**¶**^ **Change in BMI kg/m2 (95% CI)**	0.0 (-0.1, 0.1)	0.1 (-0.2, 0.4)	0.1 (-0.1, 0.2)	reference
** **p-value**	0.881	0.403	0.279	
**†** **Mean Waist circumference(95% CI)**	97.2 (93.2, 101.3)	96.2 (95.1, 97.2)	95.0 (94.7, 95.2)	95.3 (93.2, 97.5)
^**¶**^ **Change in waist circumference cms (95% CI)**	0.1 (-0.6, 0.8)	-0.4 (-0.9, 0.1)	-0.4 (-0.8, 0.1)	reference
**p-value**	0.759	0.104	0.090	
**†** **Mean LDL-cholesterol(95% CI)**	103.1 (98.1, 108.2)	106.7 (101.3, 112.2)	105.5 (96.5, 114.5)	110.8 (106.4, 115.1)
^**¶**^ **Change in LDL–cholesterol mg/dl (95% CI)**	-2.7 (-4.8 to -0.6)	-0.3 (-1.6 to 1.0)	0.0 (-1.8 to 1.9)	reference
**p-value**	0.012	0.642	0.977	
**†** **Mean CVD Risk score(95% CI)**	0.22 (0.21, 0.23)	0.24 (0.23, 0.26)	0.27 (0.23, 0.30)	0.26 (0.25, 0.27)
^**¶**^ **Change in CVD Risk score (95% CI)**	-0.006 (-0.021 to 0.007)	-0.003 (-0.010 to 0.004)	-0.002 (-0.008 to 0.004)	reference
**p-value**	0.369	0.414	0.474	
**Current Tobacco use at follow-up % (95% CI)**	13.5 (8.9, 19.4)	14.7 (9.6, 21.3)	8. 9 (4.7, 15.0)	17.5 (12.0, 24.3)
^**¶**^ **Change in current tobacco use: risk ratio(95% CI)**	1.14 (0.83 to 1.56)	1.10 (0.89 to 1.35)	0.93 (0.74 to 1.17)	reference
**p-value**	0.430	0.392	0.529	
**†** **Mean METs(95% CI)**	1101.8 (753.5, 1450.1)	910.95 (649.0, 1172.9)	798.43 (244.5, 1353.4)	916.5 (671.0, 1162.0)
^**¶**^ **Change in METs (95% CI)**	-313.14(-984.38 to 358.10)	-464.55(-1268.02 to 338.92)	-564.35(-1251.45 to 122.76)	reference
**p-value**	0.361	0.257	0.107	

* GP = general practitioner; HHE = home health education.

**†** Mean follow-up levels are based on individuals with available readings at 7 years post trial follow-up (n = 740)

^**¶**^ Change is the difference between baseline minus follow-up with negative sign indicating a decline; it was adjusted for clustering, age, gender, diabetes status, and baseline measurements

** P values indicate comparison with usual care (reference)

Adjusted risk ratios computed from adjusted marginal probabilities using logistic generalized estimating equations model (Santos, Carlos AST, et al. "Estimating adjusted prevalence ratio in clustered cross-sectional epidemiological data." BMC medical research methodology 8.1 (2008): 80).

### Sensitivity and Subgroup Analysis

The results of sensitivity analysis after imputing missing values (Table B in S1 File. Sensitivity Analysis*: Blood Pressure Levels), as well as analysis stratified by diabetes status at baseline yielded results consistent with the findings reported above (Table C in S1 File. Outcomes- Difference in Change in Blood Pressure Levels in Subgroups with and without Diabetes).

In the analysis restricted to individuals who responded at 7 year follow-up (n = 740), the self-reported adjusted risk ratio of anti-hypertensive medication use was significantly greater (1.14 95% CI, 1.02 to 1.28) in the combined HHE plus trained GP compared to no intervention group (Table D in S1 File. Adherence to Anti-hypertensive mediations).

## Discussion

After 7 years of follow-up, and 5 years after cessation of the COBRA trial, systolic BP levels among those randomised to the benefit of combined HHE plus trained GP intervention were significantly lower (2.1 [4.1–0.1]) compared to those randomised to usual care. The HHE-only group had 1 (2.2–0.2) mm Hg greater reduction in systolic BP than usual care. The combined intervention also led to greater reduction in LDL-cholesterol by 2.7 (4.8–0.6) mg/dl than usual care. A trend towards a reduction in all-cause mortality by about 20% was observed among those randomized to the combined interventions group, albeit the confidence intervals were wide. Given the small number of deaths, estimates of mortality outcomes are inevitably imprecise. The persistence of benefit on BP and additional gains from LDL-cholesterol reduction that was significant despite cessation of intervention for 5 years highlight the potential for scaling-up simple strategies for cardiovascular risk reduction.

Results from trials and meta-analysis of studies in adults with uncontrolled hypertension clearly demonstrate a strong relationship between BP lowering and CVD risk reduction- even a 1 mm Hg reduction in systolic BP decreased the risk of stroke by 5%. [15] This translates to a large absolute benefit when considered at the population level. For example, a reduction in systolic BP of 2 mm Hg at the general population in India over 30 years has been projected to avert 7% and 9% of deaths from myocardial infarction and stroke, respectively. [16] Clearly, the clinical implications of our findings from a public health perspective are substantial.

Previous trials on hypertension prevention and management that have included a greater emphasis on behavioral components and suggest a beneficial legacy effect. For example, the benefit appeared to increase in post trial surveillance of the Trials of Hypertension Prevention—-TOHP, a "pure" behavioral intervention trial. [4] By contrast, a diminution of effect was observed in trials of focusing primarily on antihypertensive medications rather than long-term behavior change (e.g. the Antihypertensive and Lipid Lowering to Prevent Heart Attack Trial—-ALLHAT) [17]

COBRA was designed to support the behavior change of the participants and the providers- thus long term benefits were expected. Moreover, developing country populations differ markedly in terms of level of education (50% literate in Pakistan) and diet (high salt consumption at 8 grams/day), than those in the industrialized world where most trials are conducted. [18] Similarly, GPs in Pakistani communities would be expected to have some deficiencies in knowledge and practices—which were identified. [19] Studies such as INTERHEART show a much higher case fatality from CVD in LMICs than in high income countries. [20] Thus, there is greater scope, and greater likelihood of considerable benefit, from interventions such as ours which target both individual knowledge and health behaviours, and health care provider skill base. The analysis restricted to subjects with available information at follow-up showed improved adherence to antihypertensive medications in the combined HHE plus GP group which could be a potential mediator. Furthermore, while dietary sodium and potassium were not estimated in COBRA, reduction in salt intake and increase in consumption of fruit and vegetables were important components of the HHE delivered by the CHW, and could have potentiated the impact of the intervention. [21] Likewise, our findings suggest that other pleotropic benefits of interventions in addition to BP reduction can contribute to some of the benefit such as on LDL-C reduction.

Our post trial findings have limitations. First, in addition to a high mortality rate, 28% (n = 290) of the targeted cohort (n = 1030) could not be tracked for BP measurement at follow-up. However, the GEE models used in the main analysis relies on the population average of all available readings while accounting for missing values at random. Moreover, our sensitivity analyses using a variety of strategies for imputing missing data yielded consistent results. Second, our study was under-powered to detect a difference in all-cause mortality. We tried to capture information on CVD morbid events, and to classify causes of death, but that the data were too unreliable to do so. However, relatively small BP differences have been shown to translate into CVD morbidity and mortality differences. [22] Since CVD accounts for about a third of all deaths among adults in South Asia, [23] even small reductions in BP at a population level would be worthwhile. It is important to note that mortality benefit in the Multiple Risk Factor Intervention Trial (MRFIT) was apparent only during post trial monitoring, despite a significant reduction in BP during the 7 year in-trial phase. [24] Third, as lipids were not measured at 2 years it is not possible to determine whether the beneficial impact of combined HHE plus trained GP intervention on LDL-lowering was more or less pronounced at 7 years than during 2 years of active intervention. Fourth, we did not measure job stress previously reported to be a component of cardiovascular risk in South Asians. [20] Finally, we were unable to determine which components of the packaged intervention contribute to the long-term benefit on systolic BP and LDL-cholesterol. However, analysis restricted to individuals alive at post-trial follow-up indicate that the increase in self-reported adherence to antihypertensive medications was greater in the combined intervention group than in the usual care group Table A in S1 File. Although speculative, it is conceivable that the benefit of combined HHE plus trained GP intervention on BP, mortality, and LDL-cholesterol would be much more pronounced had the simple intervention continued throughout the 7 years of follow-up, or longer than the initial 2 years.

Our findings underscore the overall effectiveness and durability of relatively short-term HHE and trained GP intervention in lowering cardiovascular risk in a native urban South Asian population in the mid-to long term. These findings have implications for other LMICs with similar disease burden and health systems infrastructure e.g. China, Latin America, and Africa where private GPs are the front-line care providers for ambulatory care, and non-physician health workers routinely provide door to door preventive maternal and child care services providing an opportunity for piggybacking HHE. [25–27] We now show that such a practical model based on existing health systems infrastructure is likely to have a long term benefit on BP lowering and possibly mortality.

The main strengths of our study are 1) our ability to track long term information on a representative cohort of hypertensive adults at 7 years in a low- middle- income country of 180 million; 2) using standardized measures of BP and lipids on participants during baseline and follow-up period simultaneously in the clusters to account for any potential bias due to seasonal variations in BP 3) intention-to-treat analysis preserving original randomization; and 4) sensitivity analysis including multiple imputation demonstrated consistent results.

In conclusion, our findings indicate that investment in short-term training of doctors in primary care and home-based education can generate long-term benefits on a pivotal risk factor for cardiovascular disease. Additional refresher training and home education may reinforce these effects and would require evaluation. These simple strategies are implementable and relevant to other LMICs.

## Supporting Information

S1 CONSORT Checklist CONSORT checklist for COBRA trial. (DOC)

S1 File Table A, Socio-demographic characteristics of Responders versus Non-responders for Blood Pressure level at post trial follow-up by Randomized Groups. Table B, Sensitivity Analysis: Blood Pressure Levels. Table C, Outcomes- Difference in Change in Blood Pressure Levels in Subgroups with and without Diabetes. Table D, Adherence to Anti-hypertensive mediations. (DOCX)

S2 File COBRA dataset and codebook. (ZIP)

S1 Protocol Study protocol for COBRA trial. (DOC)
