# Supplementary material for: Control of Blood Pressure and Risk Attenuation: Post Trial Follow-Up of Randomized Groups
Source: PLoS One. 2015 Nov 5;10(11):e0140550. doi: 10.1371/journal.pone.0140550 (PMC4634976; doi:10.1371/journal.pone.0140550)
Supplement: S1 File — (DOCX) [file pone.0140550.s002.docx]

**Table A. Socio-demographic characteristics of Responders versus Non-responders for Blood Pressure level at post trial follow-up by Randomized Groups**

| **Responders (n = 740) with BP measurement at 7 years (Yes) versus those who died (n = 311) (No) during follow period** | | | | | | | | | | | | | |
| --- | --- | --- | --- | --- | --- | --- | --- | --- | --- | --- | --- | --- | --- |
| **Characteristics** | **HHE and GP Group**  **n =332** | | | **HHE only Group**  **n = 348** | | | | **GP only Group**  **n = 335** | | | **Usual Care Group**  **n =326** | | |
|  | Yes | No | P value | Yes | | No | P value | Yes | No | P value | Yes | No | P value |
| **Age mean (SD)** | 51.3±9.7 | 66.7±12.3 | <0.001 | 50.7±10.1 | | 60.3±11.8 | <0.001 | 53.6±9.9 | 61.8±12.0 | <0.001 | 51.1±9.3 | 61.7±11.0 | <0.001 |
| **Men, n (%)** | 62 (29.0) | 28 (41.8) | 0.050 | 64 (34.0) | | 35 (44.9) | 0.096 | 57 (37.5) | 47 (52.2) | 0.025 | 63 (33.9) | 31 (40.8) | 0.289 |
| **Systolic BP** | 148.1±23.7 | 157.4±26.5 | 0.007 | 149.7±20.5 | | 162.5±24.7 | <0.001 | 150.1±24.3 | 158.0±27.4 | 0.021 | 149.7±25.0 | 162.5±25.5 | <0.001 |
| **Diastolic BP** | 91.3±12.1 | 91.1±15.1 | 0.928 | 93.1±11.4 | | 95.0±12.8 | 0.232 | 92.6±13.4 | 91.3±15.9 | 0.485 | 94.8±13.0 | 96.3±13.4 | 0.389 |
| **Diabetes, n (%)** | 44 (21.2) | 24 (48.0) | 0.003 | 42 (23.5) | | 33 (44.6) | 0.001 | 44 (29.5) | 37 (44.6) | 0.021 | 52 (28.6) | 28 (39.4) | 0.095 |
| **Responders (n = 740) with BP measurement at 7 years (Yes) versus those who were not reportedly dead but loss to follow-up (n = 290) (No)** | | | | | | | | | | | | | |
|  | **HHE and GP Group**  **n =332** | | | | **HHE only Group**  **n = 348** | | | **GP only Group**  **n = 335** | | | **Usual Care Group**  **n =326** | | |
|  | Yes | No | P value | | Yes | No | P value | Yes | No | P value | Yes | No | P value |
| **Age** | 51.3±9.7 | 50.1±9.7 | 0.418 | | 50.7±10.1 | 50.5±9.9 | 0.885 | 53.6±9.9 | 51.8±9.8 | 0.166 | 51.1±9.3 | 50.4±9.8 | 0.592 |
| **Men, n (%)** | 62 (29.0) | 22 (43.1) | 0.051 | | 64 (34.0) | 34 (41.5) | 0.244 | 57 (37.5) | 34 (36.6) | 0.882 | 63 (33.9) | 24 (37.5) | 0.599 |
| **Systolic BP** | 148.1±23.7 | 138.9±18.7 | 0.010 | | 149.7±20.5 | 147.6±18.1 | 0.427 | 150.1±24.3 | 150.9±27.1 | 0.823 | 149.7±25.0 | 151.4±25.6 | 0.652 |
| **Diastolic BP** | 91.3±12.1 | 87.8±11.5 | 0.057 | | 93.1±11.4 | 93.8±11.1 | 0.651 | 92.6±13.4 | 92.6±14.6 | 0.999 | 94.8±13.0 | 95.8±13.2 | 0.586 |
| **Diabetes, n (%)** | 44 (21.2) | 8 (16.3) | 0.449 | | 42 (23.5) | 20 (26.0) | 0.667 | 44 (29.5) | 24 (27.9) | 0.792 | 52 (28.6) | 14 (23.0) | 0.393 |

* Use of antidiabetic medications or a fasting blood glucose level _7.0 mmol/L (_126 mg/dL). Blood samples were missing for 62 participants

HHE = Home Health Education; GP = General practitioners

Total n = 1341 (responders = 740 and non-responders 601) All estimates are presented as mean ± SD unless specified

**Table B. Sensitivity Analysis*: Blood Pressure Levels**

|  | | | | |
| --- | --- | --- | --- | --- |
|  | ***HHE + Trained GP**  **β (95% CI β)** | **HHE only**  **β (95% CI β)** | **Trained GP only**  **β (95% CI β)** | **Usual Care (95% CI β)** |
| **1. Restricted to individuals who are still alive n=1030** | | | | |
| **^a^ Change in systolic BP mm Hg (95% CI)** | -2.4  (-4.2, -0.7) | -1.4  (-2.8, -0.1) | -1.3  (-2.8, 0.2) | reference |
| ***p-value*** | 0.007 | 0.035 | 0.086 |  |
| **2. Adding 1 mm Hg systolic BP to the last available BP of those with missing BP at 7 year.** | | | | |
| **^a^ Change in systolic BP mm Hg (95% CI)** | -2.5  (-4.4, -0.6) | -1.5  (-2.7, -0.3) | -0.9  (-2.7, 0.9) | reference |
| ***p-value*** | 0.008 | 0.012 | 0.344 |  |
| **3. Adding 2 mm Hg to systolic BP to the last avaiable BP of those with missing BP at 7 year.** | | | | |
| **^a^ Change in systolic BP mm Hg (95% CI)** | -2.5  (-4.4, -0.7) | -1.5  (-2.6, -0.3) | -0.9  (-2.7, 0.9) | reference |
| ***p-value*** | 0.008 | 0.012 | 0.344 |  |
| **4. BP reading of 2 year carried forward to 7 year for individuals who died (311), and intervention cluster-specific means imputed for all other missing values (n=290).** | | | | |
| **^a^ Change in systolic BP mm Hg (95% CI)** | -2.6  (-3.9, -1.3) | -1.5  (-2.1, -0.8) | -1.1  (-2.2, 0.1) | reference |
| ***p-value*** | <0.001 | <0.001 | 0.083 |  |
| **5. Overall cluster means assigned to all missing BP values at 7 year (lost to follow up (n=290) and those who died (n=311)).** | | | | |
| **^a^ Change in systolic BP mm Hg (95% CI)** | -1.4  (-2.4, -0.5) | -0.8  (-1.6, 0.1) | -0.8  ( -1.7, -0.1) | reference |
| ***p-value*** | 0.004 | 0.059 | 0.081 |  |
| **6. Multiple Imputation of all missing BP values at 7 year** | | | | |
| ***Change in systolic BP mm Hg (95% CI)** | -2.7  (-4.7, -0.6) | -1.5  (-3.5, 0.8) | -1.5  (-3.7, 0.7) | reference |
| ***p-value*** | 0.012 | 0.13 | 0.18 |  |

GP = general practitioner; HHE = home health education.

^a^Adjusting for clustering, age, gender, diabetes status, and baseline systolic BP

**Table C. Outcomes- Difference in Change in Blood Pressure Levels in Subgroups with and without Diabetes**

| **Individuals with Diabetes n=370** | | | | |
| --- | --- | --- | --- | --- |
|  | ***HHE + Trained GP**  **β (95% CI β)**  **(n=76)** | **HHE only**  **β (95% CI β)**  **(n=95)** | **Trained GP only**  **β (95% CI β)**  **(n=105)** | **Usual Care (95% CI β)**  **N=94)** |
| **^a^ Change in systolic BP mm Hg (95% CI)** | -3.0  (-4.6, -1.4) | -2.7  (-4.4, -1.1) | -0.1  (-1.5, 1.3) | reference |
| ***p-value*** | <0.001 | 0.001 | 0.916 |  |
| **^a^ Change in diastolic BP mm Hg (95% CI)** | -0.5  (-1.6, 0.6) | -0.00  (-0.1, 1.00) | -1.07  (0.7, -2.9) | reference |
| ***p-value*** | 0.346 | 0.996 | 0.242 |  |
| **Individuals without Diabetes n=909** | | | | |
|  | **HHE + Trained GP**  **β (95% CI β)**  **(n=241)** | **HHE only**  **β (95% CI β)**  (n=235) | **Trained GP only**  **β (95% CI β)**  (n=213) | **Usual Care β (95% CI β)**  (N=220) |
| **^a^ Change in systolic BP mm Hg (95% CI)** | -1.9 (-4.1, 0.2) | -0.8 (-2.3, 0.7) | -2.2 (-3.6, -0.8) | reference |
| ***p-value*** | 0.080 | 0.312 | 0.003 |  |
| **Change in diastolic BP mm Hg (95% CI)** | -0.1 (-1.6, 1.4) | -0.1 (-1.6, 1.4) | -0.8 (-2.2, 0.2) | reference |
| ***p-value*** | 0.952 | 0.865 | 0.296 |  |

*GP = general practitioner; HHE = home health education.

^a^Adjusted for clustering, age, gender, and baseline systolic blood pressure, or diastolic blood pressure.

**Table D. Adherence to Anti-hypertensive mediations**

|  | **HHE + Trained GP risk ratio (95% CI β)**  **N=213** | **HHE only**  **risk ratio**  **(95% CI β)**  **n=188** | **Trained GP only**  **risk ratio**  **(95% CI β)**  **n=152** | **Usual Care**  **risk ratio**  **(95% CI β)**  **n=186** |
| --- | --- | --- | --- | --- |
| **Receiving anti-hypertensive Medications at 7 year follow-up n (%)** | 126 (59) | 95 (50) | 82 (54) | 98 (53) |
| **^a^ Change in anti-hypertensive medication use Risk Ratio (95% CI)** | 1.14  (1.02, 1.28) | 1.09  (0.93, 1.27) | 1.05  (0.85, 1.29) | reference |
|  |  |  |  |  |
| ***p-value*** | 0.022 | 0.298 | 0.675 |  |

GP = general practitioner; HHE = home health education.

Adherence defined as self-reported use of anti-hypertensive medications.

^a^Adjusted for clustering, age, gender, and use of anti hypertensive medication at baseline.
