# Supplementary material for: Control of Blood Pressure and Risk Attenuation: Post Trial Follow-Up of Randomized Groups
Source: PLoS One. 2015 Nov 5;10(11):e0140550. doi: 10.1371/journal.pone.0140550 (PMC4634976; doi:10.1371/journal.pone.0140550)
Supplement: S1 Protocol — (DOC) [file pone.0140550.s004.doc]

***Study Protocol***

**Control of Blood Pressure and Risk Attenuation**

**(COBRA)**

**Post-trial Follow-Up**

*Principal Investigator*

*Professor Tazeen H. Jafar*

**Department of Community Health Sciences**

**Aga Khan University**

**Funded by**

**Wellcome Trust, UK**

**January, 2012**

Contents

1 INTRODUCTION [3](#__RefHeading___Toc306641674)

1.1 Background [3](#__RefHeading___Toc306641675)

1.2 Work that has led up to project [3](#__RefHeading___Toc306641676)

1.2.1 Cardiovascular disease in South Asians [3](#__RefHeading___Toc306641677)

1.2.2 Trial in Karachi [5](#__RefHeading___Toc306641678)

1.2.3 Main Trial findings – [7](#__RefHeading___Toc306641679)

1.3 Rationale [8](#__RefHeading___Toc306641680)

1.3.1 Rationale for following up BP and CVD morbidity and mortality: [8](#__RefHeading___Toc306641681)

1.3.2 Rationale for assessing behavioural risk factors [9](#__RefHeading___Toc306641682)

1.3.3 Rationale for assessing clinical cardiovascular risk factors [9](#__RefHeading___Toc306641683)

1.3.4 Rationale for sub-clinical CVD outcome – LV mass and diastolic dysfunction [9](#__RefHeading___Toc306641684)

1.3.5 Rationale for ancillary studies [10](#__RefHeading___Toc306641685)

1.4 Aims of the project [11](#__RefHeading___Toc306641686)

1.5 Hypotheses: [12](#__RefHeading___Toc306641687)

1.6 Ancillary Studies: [12](#__RefHeading___Toc306641688)

2 METHODS [13](#__RefHeading___Toc306641689)

2.1 Assessment of left ventricular mass and diastolic dysfunction: [13](#__RefHeading___Toc306641690)

2.2 Assessment of morbid and mortal events: [14](#__RefHeading___Toc306641691)

2.3 Ancillary Study- Survey of General Practitioners: [14](#__RefHeading___Toc306641692)

2.4 Outcome Measures [15](#__RefHeading___Toc306641693)

2.4.1 Research Team [15](#__RefHeading___Toc306641694)

2.4.2 Training of Field team [15](#__RefHeading___Toc306641695)

2.4.3 Pre-testing [15](#__RefHeading___Toc306641696)

2.4.4 Data Collection [16](#__RefHeading___Toc306641697)

2.5 Data Management [17](#__RefHeading___Toc306641698)

3 PLAN OF ANALYSIS: [18](#__RefHeading___Toc306641699)

3.1 Primary & Secondary Outcomes: [18](#__RefHeading___Toc306641700)

3.2 Ancillary Analysis: [18](#__RefHeading___Toc306641701)

3.3 Ethical Considerations [19](#__RefHeading___Toc306641702)

4 TIME LINE: [21](#__RefHeading___Toc306641703)

5 REFERENCES: [22](#__RefHeading___Toc306641704)

#

# INTRODUCTION

## Background

Cardiovascular disease (CVD) has become the leading cause of mortality worldwide, accounting for 30% of deaths annually in low and middle income countries.[^1^](#_ENREF_1) In South Asia, high rates of CVD are observed at a younger age than in other countries, causing a greater loss of productive life years, and severe economic consequences. High BP confers the highest attributable risk to death and disease associated with CVD.[^1^](#_ENREF_1) The National Health Survey of Pakistan (1990-1994) conducted by the Pakistan Medical & Research Council under the technical guidance of US Centres for Diseases Control & Prevention provided alarmingly high estimates of the prevalence of hypertension, affecting one in three adults aged 45 years or older across all socioeconomic strata in both urban and rural areas of all provinces in Pakistan.

We conducted the Wellcome Trust funded *Control of Blood Pressure and Risk Attenuation* (COBRA) trial (2004 to 2007) in Karachi, Pakistan, to test the effectiveness of two strategies on blood pressure (BP) levels; the first was a population based approach consisting of family based home health education (HHE) delivered by trained community health workers to improve population level health literacy and behaviours; the second was a high risk approach using trained general practitioners (GP) to optimally manage hypertension. Interventions were tested alone, and in combination over 2 years. We hypothesized that HHE would be more effective than none, trained GPs more effective than usual care provided in Karachi, and that there may be an additional benefit of the combined intervention.

In children and young adults, the age related rise in systolic BP was significantly attenuated in those randomized to HHE versus no HHE.[^2^](#_ENREF_2) In those at high risk (40+ with hypertension), we observed that the combined strategies (HHE plus trained GP) had the most marked beneficial impact on BP.[^3^](#_ENREF_3) However, the trial was of short duration and it is not known whether the benefit of interventions on BP observed at 2 years would be sustained in the medium term in the post-trial period. We now wish to conduct 4 year post trial follow-up of all the COBRA trial participants, with intensive efforts to contact all those lost to follow-up, to determine the sustained impact of our interventions on a) BP levels and cardiovascular morbidity and mortality; b) behavioural risk factors (tobacco use, diet, weight, physical activity); and clinical risk factors (plasma glucose, lipids and albuminuria) for CVD; and sub-clinical cardiovascular outcomes (left ventricular mass and diastolic dysfunction) in the trial participants. We will also assess adherence to hypertension treatment guidelines by the practitioners, and influence of BP and antihypertensive therapy on quality of life of these subjects. In addition, the relationship of repeated BP measurements over time with clinical and sub-clinical cardiovascular outcomes will be examined.

Thus, the post trial follow-up of COBRA participants will provide valuable insights on the presence of sustained benefit of interventions after their discontinuation. This information is key for up-scaling a national programme modelled on similar interventions for Pakistan, which has been proposed to the Federal Ministry of Health, and is potentially scalable in neighbouring countries. The study would also be novel in advancing the knowledge on the relationship of BP with robust clinical and sub-clinical cardiovascular outcomes in Indo-Asians.

## Work that has led up to project

### Cardiovascular disease in South Asians

Mortality from CVD in South Asian populations is one of the highest in the world, being 1.5 fold greater than populations of European origin. In South Asia, high rates of CVD are observed at a younger age than in other countries, causing a greater loss of productive life years due to premature morbidity and mortality.[^1^](#_ENREF_1),[^4^](#_ENREF_4)

*Cardiovascular disease in Pakistan*

We have confirmed the large burden of CVD in urban Pakistan, where it affects almost a quarter of both men and women aged 40 years and above.[^5^](#_ENREF_5),[^6^](#_ENREF_6) The burden of risk factors was also elevated. Based on nationally representative estimates, in individuals aged 45 years and above, 10% had diabetes, 18% had high cholesterol, 32% used tobacco, and 32% were overweight or obese.[^11^](#_ENREF_11)

A predisposition to high BP was apparent during childhood. The analysis of over 5000 children aged 5-14 years surveyed during the NHSP 1990-1994 suggested that body-mass adjusted BP levels were higher in Pakistani children compared to white children in the United States.[^12^](#_ENREF_12) Hypertension prevalence in adults aged 40 years or older was especially high at 33% in both urban and rural areas, affecting affluent individuals as well as the impoverished majority- 2/3^rd^ of the Pakistani population live on less than $2 per day.[^14^](#_ENREF_14)

Results of the NHSP 1990-1994 also indicate that on an average a Pakistani visits a health provider 5 times annually, which is fairly high frequency of contact. Despite that, 70% of adults with high BP were unaware of their condition and only 35% reported ever having BP measured. The lack of screening for BP was worse in the less educated. These data also underscore that both poverty and lack of education are strong social determinants of hypertension, thereby enhancing its vulnerability among the disadvantaged in Pakistan.[^10^](#_ENREF_10) Consequently, less than 3% of all persons with hypertension had adequately controlled BP.[^8^](#_ENREF_8) Despite the high burden, we identified serious deficiencies in the knowledge and practices of primary care practitioners regarding the management of this condition, for example recommending sedatives as first line therapy.[^15^](#_ENREF_15) The 2005 Pakistan Demographic Survey (PDS), conducted by the Pakistan Federal Bureau of Statistics, indicates that CVD accounted for 23% of all crude deaths and 33% of age standardized deaths. Further, about a quarter of all deaths recorded in the PDS 2005 were in persons aged 15-59 years, and NCDs were responsible for 65% of these deaths. Notwithstanding the limitations of existing vital registration system, these estimates are consistent with regional data and indicate a substantial loss of human capital with grave implications on national economy.[^16^](#_ENREF_16)

**Sampling Frame**

City District Government of Karachi

4200 low to middle income clusters

178 union councils (5000 clusters)

18 Towns

800 High income clusters excluded

12 clusters, randomly selected from the block

350 contagious blocks mapped

### Trial in Karachi

From 2004 to Dec 2007, we conducted a Wellcome Trust funded 2x2 factorial cluster RCT (ref no 070854), intervention trial of BP lowering. Using multistage cluster sampling techniques, 12 clusters were randomly selected from middle to low income areas in Karachi. The total number of subjects aged 5 years or above in the study clusters was 17,500. Out of these, one individual in the age groups of 5-14 and 15-39 years was randomly selected from each of the households (a household is defined as individuals in a house sharing one kitchen) and all those aged 40 and over were also invited for interview and measurements. We compared a population approach using home health education (HHE) for households in children and young adults, and a high risk approach for adults aged 40 plus with hypertension (see study diagram). To ensure scalability, both approaches were modelled on the existing healthcare infrastructure in Pakistan:

*Population Approach:* Twelve clusters (a cluster is a defined geographical area containing HWscommunity lady health workers delivered at 3-monthly intervals, or no home health education (control).

*High-Risk Approach:* Adults in these households aged ≥ 40 years who were hypertensive were included in a comparison of usual versus care from GPs trained to a standardized protocol.

#### Interventions:

*Home Health Education:* Six community health workers (CHW) (one for each cluster) were trained over six weeks in methods of conveying standardized health education messages to all households in clusters assigned to HHE, using behaviour changing communication strategies. The health messages included advice on the importance of engaging in moderate physical activity; maintaining normal body weight; reducing salt intake; maintaining adequate intake of potassium; and consuming a diet rich in fruit, vegetables, and low-fat dairy products and reduced in saturated and total fat, and smoking cessation.[^3^](#_ENREF_3) The importance of achieving BP goal and adherence with medication and physician follow-up were emphasized. The first HHE session, lasting 90 minutes, was held at a time when all members of the household could be present. Follow up reinforcement visits of 30 minutes were performed at 3 monthly intervals. This intervention was modelled on the existing lady health workers (LHWs) programme in Pakistan which provides door-to-door basic maternal and child care services to 80 million people in the country at an estimated per capita cost of $0.75, inclusive of human resources, training and other operational expenses.[^17^](#_ENREF_17) We reasoned that if successful the health gains are likely to outweigh the nominal marginal cost of adding our modest proposed strategy onto the existing platform of LHWs in Pakistan.

*General Practitioner Education:* All GPs in the six study areas assigned to this intervention were invited for training, with the realistic aim to train at least two-thirds of all GPs from each area. The training of community GPs instead of specialised physicians was again to ensure a sustainable programme. The training was a one day session focused on standard treatment for the management of hypertension, based on the seventh report of the Joint National Committee (JNC 7) and the Fourth Working Party of the British Hypertension Society guidelines modified for the Indo-Asian population.[^18^](#_ENREF_18),[^19^](#_ENREF_19),[^3^](#_ENREF_3) Components of the course included non-pharmacological (diet, exercise, weight loss, smoking cessation) and pharmacological interventions; prescribing low cost and appropriate generic drugs; preferential use of single dose drug regimens; scheduled follow-up visits guided by level of BP; stepped care approach for titration of drugs to achieve target BP levels; and satisfactory consultation sessions for patients, with explanations of treatment and use of appropriate communication strategies.

Study Diagram

12 Clusters

n = (7,166)

Total aged 40 years and over n = (3143),

Total aged 15-39 years n = (2348),

Total aged 5-14 years n = (1675)

Household Health Education (HHE) n = (1313)

Aged 40 years and over n = (1,632),

Aged 15-39 years n = (1,175),

aged 5-14 years n = (833)

(Height, Weight, Blood Pressure, Waist Hip ratio,

Food Frequency Questionnaire, Tobacco,

Physical Activity)

Age > 40 years n = (1,511)

((FPS, lipids, ACR, ECG) ECG)

)

Ineligible for other components of the study

Age > 40 years n = (1,632)

(FPS, lipids, ACR, ECG)

Age > 40 years

with hypertension

n = 680

(FBS, lipids, ACR, ECG, Echo)

)

Usual Care

Hypertension Management

Special Care

Hypertension Management

6 Clusters

6 Clusters

Usual Care

Hypertension Management

Special Care

Hypertension Management

n = 348

n = 332

n = 326

n = 335

+ HTN

+ HTN

No HTN

No HTN

Age < 40 years

No Household Health Education (HHE) n = (1337)

Aged 40 years and over n = (1,511),

aged 15-39 years n = (1,173),

aged 5-14 years n = (842)

(Height, Weight, Blood Pressure, Waist Hip ratio,

Food Frequency Questionnaire, Tobacco,

Physical Activity)

Age > 40 years

with hypertension

n = 661

((FBS, lipids, ACR, ECG, Echo)

HTN=hypertension, FBS= fasting blood sugar, ACR= urine albumin to creatinine ratio, Echo=Echocardiography

### Main Trial findings –

***Results of the community and high risk approach have been published*** [^2^](#_ENREF_2),[^3^](#_ENREF_3) ***Key findings are highlighted here.***

#### Children and young adults

Of the 4023 participants aged 5 to 39 years recruited at baseline, follow-up data were available on 72% at 2 years. Using the intention-to-treat principle (cluster specific mean follow-up blood pressures were used for those not followed up), there was evidence that the intervention was associated with beneficial changes in systolic BP (adjusted for age, sex, and baseline BP).

**Change in blood pressure levels from baseline to follow up by intervention allocation in children and young adults (n=4023)**

| **Blood pressure**  **(mm Hg)** | *HHE  n=2008 | No HHE  n=2015 | **p-value |
| --- | --- | --- | --- |
| **†**Change in SBP (95% CI) | 0.1 (-0.3 to 0.5) | 1.5 (1.1 to 1.9) | 0.02 |
| **††**Change in DBP (95% CI) | 0.6 (0.3 to 0.9) | 2.1 (1.8 to 2.4) | 0.002 |

*HHE= Home Health Education;

**†**Adjusted for clustering, age, sex and baseline SBP.

**††**Adjusted for clustering, age, sex and baseline DBP.

** p-value is for difference in change in BP between the two groups

#### High risk cohort

Of the 1341 participants aged 40 years and above with hypertension (≥140 mmHg systolic or ≥ 90 mmHg diastolic or already on treatment) recruited at baseline, follow-up data were available on 78%. The age, sex, baseline BP-adjusted decline (95% CI) in systolic BP was significantly more pronounced in the HHE plus trained GP group (10.8 (8.9 to 12.8) mm Hg) as compared to trained GP alone, HHE alone, or no intervention groups 5.8 (3.9 to 7.7) mm Hg, in each (p<0.001). The interaction between the main effects of trained GP and HHE on the primary outcome approached significance (interaction p=0.004 in intention to treat and p=0.04 in per protocol analysis).[^3^](#_ENREF_3)

**Change in Systolic Blood Pressure Levels among Randomized Groups (n=1341)**

| ***Treatment Group** | **n** | **†Decline in SBP in mm Hg (95% CI)** | **‡p-value** |
| --- | --- | --- | --- |
| HHE + Trained GP | 332 | 10.8 (8.9, 12.8) | < 0.001 |
| HHE only | 348 | 5.6 (3.7, 7.4) |
| Trained GP only | 335 | 5.6 (3.7, 7.5) |
| No Intervention | 326 | 5.8 (3.9, 7.7) |

*HHE= Home Health Education; GP= General Practitioner

†Adjusted for clustering, age, sex and baseline SBP. Interaction p for trained GP and HHE=0.004

‡ p-value for decline in BP among intervention groups

In a nested sub-study within the high risk cohort, we also observed that GP training enhanced adherence to anti-hypertensive medications in the short-term.[^20^](#_ENREF_20) This finding is consistent with the improved knowledge score of the GPs immediately after the training session.[^3^](#_ENREF_3)

#### Morbid and Mortal Events

Information on cardiovascular morbid events requiring hospitalization were collected at 6 monthly intervals and adjudicated by cardiologists and neurologists masked to randomization status, using guidelines recommended by the American Heart Association.[^21^](#_ENREF_21) Data on mortality was tracked (explained below). Deaths due to myocardial infarction, coronary revascularization, heart failure or stroke were categorized as cardiovascular deaths. During 2 years of follow-up, there were 231 composite events (cardiovascular events leading to hospitalization or all-cause mortality), 212 deaths of which 95 were due to cardiovascular causes. While the study was not powered for hard outcomes, preliminary analyses revealed an age and sex adjusted hazard ratio (95% CI) for all cause mortality for HHE versus no HHE of 0.92 (0.59-1.43), and for trained GP versus usual care GP of 0.73 (0.47-1.14). No interaction was detected between HHE and GP training on either the composite outcomes or all-cause mortality.

## Rationale

### Rationale for following up BP and CVD morbidity and mortality:

The main trial was designed to assess the impact of family based HHE delivered every three months to the households in the randomized clusters. We found that this strategy was beneficial at the end of 2 years in lowering BP. However, we could not draw conclusions on the post trial effect of our intervention, whether they are lost or sustained at the same or an augmented level. Long term follow-up of trial participants after discontinuation of intervention in other studies have yielded conflicting results. For example, in the United Kingdom Prospective Diabetes Study (UKPDS) the benefit of tight control of BP on mortality was lost at 10 years.[^22^](#_ENREF_22) There are concerns that life style modifications may be difficult to sustain in the absence of continuous re-enforcement. Even in dietary interventions with overall success from nutrition counselling, subjects tend to regain weight after an initial decline.[^23^](#_ENREF_23) However, the characteristics of developing country populations can be substantially different in terms of level of education (50% literate in Pakistan) and diet (high salt consumption at 8 grams/day), than those in the industrialized world where most trials are conducted.[^24^](#_ENREF_24),[^13^](#_ENREF_13) The same applies to GPs in the communities, in whom serious deficiencies in knowledge and practices were identified.[^15^](#_ENREF_15) Thus, part of the success of our strategies could be due to the greater scope for improvement in knowledge and behaviour in the study population. Therefore, we expect most lifestyle changes to be retained and reflected in sustained benefit on BP. The post trial benefit of life style intervention on diabetes prevention in China supports our contention although admittedly impacts on a different outcome, hypertension, could differ.[^25^](#_ENREF_25) Although there is strong evidence to expect substantive benefit in CVD outcomes from reduction in BP[^26^](#_ENREF_26) considerable dissociation between the impact on BP and cardiovascular morbidity and mortality has been observed in some studies. For example, in the Antihypertensive and Lipid-Lowering to Prevent Heart Attack Trial (ALLHAT), a federally funded study, the combined CVD outcomes were disproportionately greater in doxazosin versus chlorthalidone arm (4-year rates, 25.45% versus 21.76%; Relative Risk , 1.25; 95% CI, 1.17-1.33; P<0.001) than expected from a 2 mmHg difference in systolic BP.[^27^](#_ENREF_27) More recently, the Avoiding Cardiovascular Events through Combination Therapy in Patients Living with Systolic Hypertension (ACCOMPLISH) trial was stopped prematurely at a median follow-up of 3 years when a significant difference was observed in CVD events: 9.6% in the benazepril–amlodipine group 11.8% in the benazepril-hydrochlorothiazide group; a relative risk reduction of 19.6% (p<0.001); despite no difference in BP between the two groups.[^28^](#_ENREF_28) At the same time, it is also important to note that mortality benefit in the Multiple Risk Factor Intervention Trial (MRFIT) was apparent only during post trial monitoring, despite significant reduction in BP during the 7 year in-trial phase.[^29^](#_ENREF_29) Thus, other pleotropic benefits of interventions in addition to BP reduction can contribute to some of that benefit. Finally, as mentioned above, successes or failures of the developed countries may not be exportable to developing ones, where significant differences exist in the population characteristics and healthcare systems.[^30^](#_ENREF_30) Therefore, post trial follow up of COBRA is needed to assess any benefit of our interventions after their discontinuation on sustained lowering of BP, and its translation into reduction in CVD morbidity and mortality in the Pakistani population. The extended follow-up of these outcomes will also enable the determination of the relationship between the duration of BP lowering on CVD outcomes. This information will be most valuable in up-scaling strategies for BP control and CVD prevention, and designing new interventions for further improvement in Pakistan, and neighbouring countries.

### Rationale for assessing behavioural risk factors

Both the interventions in COBRA-HHE and GP training- were centred on health related behaviour modification. Whilst the study was not powered to assess changes in secondary outcomes of life style factors during 2 years, improvements in smoking and physical activity with intervention were detected in the high risk cohort.[^3^](#_ENREF_3) A longer term follow-up of all participants will allow greater power to assess the impact of the strategies on these as well as other key behavioural risk factors responsible for some of the potential benefit on BP and CVD, including obesity, dietary intake of fruit and vegetables, and use of antihypertensive medications.[^31^](#_ENREF_31)

### Rationale for assessing clinical cardiovascular risk factors

High blood glucose and high serum cholesterol are established modifiable risk factors for CVD, and confer a high attributable burden to mortality in low and middle income countries. [^32^](#_ENREF_32) [^1^](#_ENREF_1) Both types of interventions delivered during the COBRA trial included an emphasis on health related behaviour, which have been shown to improve glycemic status and lipid profiles in other studies. [^25^](#_ENREF_25) [^33^](#_ENREF_33) About 28% of hypertensive subjects in COBRA had diabetes (fasting blood sugar ≥ 7.0 mmol/L (126 mg/dL) or on antidiabetic medication), and 39% had hyperlipidemia (fasting serum cholesterol of ≥ 5.18 mmol/L (≥200 mg/dL)). Evidence on the relationship between albuminuria and CVD is accumulating. [^34^](#_ENREF_34) Lifestyle interventions have been shown to reduce albuminuria, and [^35^](#_ENREF_35) studies also indicate that risk of CVD associated with albuminuria may be modifiable. [^36^](#_ENREF_36) Therefore, we plan to obtain a fresh blood and urine sample for measurement of these risk factors. The assessment of change in plasma glucose, serum cholesterol and albuminuria from baseline to post trial follow-up by randomized group will provide useful information on a broad range of impact of interventions- which probably extend beyond just BP lowering. The findings will also provide mechanistic clues on any benefit of their role in the reduction of CVD morbidity and mortality in our study.

### Rationale for sub-clinical CVD outcome – LV mass and diastolic dysfunction

Increased left ventricular mass is established sequelae of uncontrolled hypertension, and a modifiable predictor cardiovascular morbidity and mortality. Hypertension also leads to heart failure by causing functional abnormality in relaxation of the myocardium, manifested as diastolic dysfunction. This can be present with or without concomitant increase in left ventricular mass. Studies on asymptomatic hypertensive subjects have demonstrated significant increase diastolic dysfunction assessed via measures of decreased left ventricular relaxation, increased stiffness and filling pressures.[^41^](#_ENREF_41),[^42^](#_ENREF_42)

Our previous pilot experience on echocardiographic evidence of left ventricular mass in 320 randomly selected community based subjects revealed higher LVMI in women compared to men: the mean (SD) was 75.7 (25.9) [median 72.9] g/m(2) in women and 72.0 (19.2) [median 71.1] g/m(2) in men.[^43^](#_ENREF_43) This is not entirely surprising as women in Pakistan have a greater burden of cardiovascular risk factors associated with left ventricular mass including obesity, hypertension (and less likely to be screened for it), and metabolic syndrome than men.[^6^](#_ENREF_6) However, we also observed that the increased risk in women could not be entirely explained by conventional cardiovascular risk factors. Studies on LVMI using echocardiography in Indo-Asian population are scarce. Further, we did not assess diastolic dysfunction. Thus, the proposed study provides a unique opportunity for rigorous assessment of left ventricular mass and diastolic dysfunction according to intervention status and BP lowering in women and men recruited to COBRA. The demonstration of benefit particularly in women, will underscore the importance of targeting this vulnerable segment of the population in hypertension screening and CVD prevention programmes.

### Rationale for ancillary studies

a) Rationale for quality of life measure: There is a scarcity of information on health related quality of life (HRQoL) in Indo-Asians. Studies in Western populations have shown that hypertensive patients have a significant decrease in quality of life, especially in the domains encompassing work performance, sexual function, humor, and overall sense of well being.[^44^](#_ENREF_44) These measures can improve with treatment for lowering BP, however the degree depends upon concomitant side-effects associated with therapy and their tolerance. Evidence based on the DASH diet and Treatment of Mild Hypertension Study (TOHMS) suggests benefit of non-pharmacological interventions for BP lowering on improving HRQoL. We propose to assess determinants of HRQoL, in particular the relationship of interventions, BP at baseline and follow-up and changes in BP, and use of antihypertensive medications on work performance and overall well being of the high risk COBRA participants using the SF-36 and EQ-5D questionnaires. The performance of SF-36 has been assessed in Indian migrants in Singapore, where ethnic differences were noted due to level of education.[^47^](#_ENREF_47) We will account for socio-demographic factors including educational attainment in our analysis. The knowledge regarding the relationship of HRQoL with interventions, high BP and its treatment in Indo-Asians in Pakistan is essential for understanding the full value of treatment and its comprehensive impact on society at large.

b) Survey of GPs for adherence to hypertension guidelines: A previous study by our group revealed serious deficiencies in knowledge and practices of GPs pertaining to hypertension treatment guidelines in Pakistan, which served as the basis for training GPs in the COBRA trial.[^15^](#_ENREF_15) In the latter, a total of 78 out of 110 (71%) invited GPs in the clusters assigned to trained GP intervention received the training programme. The post-test administered immediately after training session revealed significant improvement in knowledge.[^3^](#_ENREF_3) There were 130 GPs in the other clusters. Although we showed success of this intervention when coupled with HHE, it would be important to assess how much of the knowledge imparted during training of GPs, is retained and practised by the GPs in the long-term. This information will guide the frequency of future training sessions. Thus, we propose to conduct a survey of knowledge and practices of GPs in the communities randomized to interventions.

c) Predictive Relationship of BP variability with LV mass and CV morbidity and mortality: Epidemiological studies have shown that repeated measures which account for variation in BP over time offer more precise and less biased estimates of association with CVD and mortality, which are incremental to those obtained from single BP readings.,[^50^](#_ENREF_50) The variability in BP has various dimensions and is measured using different metrics - ranging from beat-to-beat variability measured with continuous BP recordings to variation in intermittent readings spread over days, weeks, months to years to continuous BP monitoring during sleep and waking hours. In the high risk cohort in COBRA, resting brachial BP was measured at baseline and 6-monthly intervals, two readings at each home visit after 5 minutes of rest between measurements. Thus, two BP readings at each of the four home visits are available during a follow-up of two years on most subjects. In the in-trial analysis of COBRA, the time-series model using all available systolic blood pressures revealed a significant benefit of combined intervention of HHE plus trained GP over no or single intervention, consistent with that based on single follow-up reading at the final 2 year visit. In addition, a trend towards enhancement of the effect of intervention on BP with time was observed (time with treatment effect interaction p=0.02 (unpublished data)). We now wish to explore the association of repeated measures of BP spread over months to years with CVD endpoints of LV mass and CVD morbidity plus all cause mortality. These analyses are likely to unmask the true impact of intervention as they relate to various components of BP and cardiovascular outcomes over time.

## Aims of the project

To determine the sustained impact of HHE and annual GP training, alone and in combination, at 7 years, including 5 years of post intervention follow-up on:

a) Primary

i) BP levels of all participants.

ii) Cardiovascular morbid events and all cause mortality in the high risk cohort.

b) Secondary

i) Behavioural risk factors including diet, physical activity, weight and tobacco use in all trial participants

ii) Clinical risk factors for CVD including plasma glucose and lipids, and albuminuria in the high risk cohort.

iii) Left ventricular (LV) mass index and diastolic dysfunction in the high risk cohort.

## Hypotheses:

We hypothesize that at 6 years of follow-up:

- Children and young adults randomized to HHE versus no HHE will continue to have lesser rise in BP.
- Hypertensive adults randomized to HHE plus trained GP intervention will continue to have greater decline in BP than single or no intervention. They will also have experience greater decline in plasma glucose, serum cholesterol and urine albumin excretions levels, and have lower left ventricular mass index and less diastolic dysfunction.

## Ancillary Studies:

- a) To determine the influence of BP and antihypertensive therapy on quality of life in the high risk cohort
- b) To evaluate the use of practice guidelines for management of hypertension among practitioners in the randomized communities.
- c) To examine the relationship between BP variability during 2 years of intervention with LV mass index and cardiovascular morbidity and mortality at 7years.

# METHODS

All participants in the trial (originally aged 5 and now 13 years or over) will be visited by trained field staff masked to randomization status. However, the individuals (children or individuals aged 15 – 39 years) not enrolled in the main study, pregnant women, bed ridden and/or mentally retarded study subjects unable to consent will be excluded. Eligibile subjects will be offered informed consent. The date and time for interview will be set at individual’s convenience after obtaining informed consent. Information on socioeconomic status assessed via household possessions and income, occupation and literacy status, diet, physical activity (modified IPAQ), and tobacco use would be collected and weight, height, waist and hip circumference would be measured. In addition, questionnaires on health related quality of life (SF-36 and EQ-5D) will be administered to all subjects aged 40 and over with hypertension after translation in Urdu and pre-testing. As collected during baseline, the names and doses of current anti-hypertensive medications will be recorded.

As during the COBRA trial, BP will be measured with a calibrated automated device, Omron HEM-737^TM^ Intellisense BP Monitor, in the sitting position after 5 minutes of rest using an appropriate sized paediatric or adult cuff, as applicable.[^51^](#_ENREF_51) BP measurements will be repeated after two weeks. Three consecutive readings will be taken at each visit, and the mean of the final two used in the analysis. The visits will be balanced across the four randomised groups and planned during the first half of the day to minimise seasonal and diurnal variation in measurements, respectively. Maximum three visits will be paid for those not present at a scheduled date and thereafter, they will be labelled as lost to follow-up.

Since one of the main aims the post-trial follow-up is to track the cohort for morbidity and mortality, a special field team will be dedicated to tracking those who were lost to follow-up during the initial 2 years or have relocated within the city of Karachi. We expect to recapture about 15% of those lost to follow-up with the assistance of neighbours, family members and community leaders residing in the study clusters. As Karachi is considered the business and economic hub of Pakistan with an annual population growth rate of 5%, the rate of out-migration is expected to be very low.[^52^](#_ENREF_52),[^53^](#_ENREF_53) Details of current residential address will be recorded for the possibility of shifting into another randomised cluster, although the chances are minimal as they represent 12 out of 5000 (0.24%) geographical communities in Karachi.

In all subjects in the high risk cohort (aged 40 years and above with hypertension), fasting blood sample will be collected for plasma glucose (Synchron Cx-7/Delta, Beckman, US) and lipid profile (Hitachi-912, Roche Japan). A fresh morning urine sample will also be collected for albumin (measured using nephelometry by the Array Systems method on a Beckman Coulter) and urine creatinine (Synchron Cx-7/Delta, Beckman, US). For external quality control on albumin measurements, AKUH laboratory routinely sends samples to Bio-Rad Laboratories Inc, Irvine, CA. Sera and urine samples will be stored at -70 freezer for possible future analysis.

A trained sonographer (gender-specific as culturally appropriate) will perform 2-D echocardiogram with a portable device for measurement of left ventricular mass and function (see 3.1.1). All assessments will be performed to a standard protocol that conforms to international standards for definitions and measurements.

## Assessment of left ventricular mass and diastolic dysfunction:

This will be done at the field site using the portable Philips CX50 imaging system by a sonographer with recommended level of training for performing echocardiograms. One sonographer routinely performing echocardiograms will undergo standardized training including didactic presentations and hands-on supervised studies.[^54^](#_ENREF_54) The exam will be performed with the subjects in semi-recumbent in the left lateral position. Left ventricular measurements will be performed using M-mode from the parasternal long axis according to the American Society of Echocardiography guidelines.[^54^](#_ENREF_54) Left ventricular internal dimension and wall thicknesses will be measured at end-diastole and end-systole by American Society of Echocardiography recommendations using a computerized review station, and then reviewed by a cardiologist certified by the U.S. National Board of Echocardiography.[^54^](#_ENREF_54) As in the LIFE Study, end-diastolic left ventricular septal and posterior wall thicknesses and internal dimensions will be used to calculate left ventricular mass by the formula: left ventricular mass = 1.04 x 0.8 [(left ventricular wall thicknesses + internal dimension) – (internal dimension)] + 0.6 g. left ventricular mass index (LVMI), calculated as left ventricular mass in grams divided by body surface area in square meters.[^39^](#_ENREF_39)

Doppler imaging: Transmitral Doppler inflow signals will be acquired and peak early diastolic velocity (E), peak late diastolic velocity (A), the E/A ratio, pulmonary vein, mitral valve deceleration time (DT), isovolumic relaxation time (IVRT) will be measured. In addition, Tissue Doppler imaging (TDI) of mitral valve annulus in the 4 chamber view at the septal and lateral walls will be performed as per guidelines of the American Society of Echocardiography, from which peak early (E’ or Ea) and peak late (A’ or Aa) diastolic mitral annular velocities will be measured.[^55^](#_ENREF_55) The E/E’ ratio, will be computed as a reflection of left atrial pressure as a measure of left ventricular diastolic function.[^56^](#_ENREF_56) In addition, patients will be categorized into grades of left ventricular diastolic dysfunction (none, grades I, II and III) based on established criteria.[^56^](#_ENREF_56) A subset of 100 subjects, stratified by sex, will be invited for a repeat measurement within one month to assess reproducibility (limits of agreement/coefficient of variation for test-re-test repeatability of LVMI and tissue Doppler parameters. Intra-observer variation in archived measurements from the same day will also be reported).[^57^](#_ENREF_57)

## Assessment of morbid and mortal events:

As during the main trial, all subjects aged 40 years and above in the cohort will be tracked for assessment of morbid CVD events requiring hospitalizations or death during the entire 6 years of follow-up. Since data from the initial 2 years were collected at 6 monthly intervals, relevant information during the last 4 years will be extracted from district-level official mortality registers in each cluster to track mortality. A standardized questionnaire on mortality or hospital admission during the last four years will be administered in a culturally sensitive fashion. In cases of death, the cause of death reported by the nearest family member will be recorded, and the WHO verbal autopsy questionnaire will be administered. Complete death records will be extracted, where available. However, some degree of uncertainty is expected with ascertainment of cause of death is expected in capturing mortality data on verbal autopsy from beyond the last one year. All those who report admission to hospital for a cardiovascular event, or a stroke like event will be subsequently evaluated by a study cardiologist or neurologist to confirm the diagnosis per recommendations of the American Heart Association and the European Society of Cardiology.[^21^](#_ENREF_21) On ethical grounds, all these patients will also be given advice regarding treatment for any complications, and prevention of future events.

## Ancillary Study- Survey of General Practitioners:

The data collectors masked to randomization status will approach all GPs practicing in the 12 randomized clusters to administer a standardized questionnaire. It will contain questions on six basic themes: a) type of practice: average number of patients seen, b) information about the GP: gender, years into practice, c) detection of high BP: whether BP is measured on all or all new patients, how many readings and when, criteria for diagnosing high BP, and whether these criteria differ for the elderly as opposed to young adults, d) evaluation of patients with high BP: whether laboratory investigations for coexisting conditions (diabetes, hyperlipidemia, kidney failure) are obtained on hypertensive patients, e) management of hypertension: patient education regarding deleterious effects of hypertension, advice on non-pharmacological measures to control hypertension, the level of BP that is targeted during treatment, and choice of antihypertensive medications, and f) types of antihypertensives used (generic or branded), g) whether antihypertensives are discontinued once BP is controlled.

## Outcome Measures

*Primary:*

In all subjects:

- Change in systolic BP from baseline to mean of two post trial follow-up visits.
- Composite of CVD morbid events and all-cause mortality.

*Secondary:*

In all subjects:

- Change from baseline to post trial follow-up in the following: body mass index (BMI), waist hip ratio, current tobacco use, total physical activity, and dietary intake of fruit and vegetables.

In the high risk cohort

- Percent change in plasma glucose (and incident pre-diabetes or diabetes defined as (fasting plasma glucose 100 mg/dl (5.5mmol/L) or more or initiation of hypoglycaemic agent)[^58^](#_ENREF_58), total and LDL cholesterol (and incident hyperlipidemia defined as total serum cholesterol >=200 mg/dl (5.18 mmol/L); LDL cholesterol >=130 mg/dl (3.37mmol/L))[^59^](#_ENREF_59), albumin excretion rate (and incident albuminuria defined as spot urine albumin to creatinine ratio of 30mg/g or greater)[^60^](#_ENREF_60) , and estimated glomerular filtration rate from baseline to post trial follow-up;
- Left ventricular mass index and E/A’ ratio at post trial follow-up.[^39^](#_ENREF_39),[^61^](#_ENREF_61)

Ancillary:

In the high risk cohort

- Overall mean score on Patient Health Related Quality of Life (HRQOL) based on 36-Item Short-Form Survey.

Data Collection and Management

### Research Team

The research team will consisted of research manager, data manager, one communication officer, two field supervisors, two data editors, one echo technologist and the 10 data collectors in pairs (five males and five females) will be assigned for data collection.

### Training of Field team

The team will be rigorously trained over a six-week period in study techniques, including performing standardized BP and anthropometric assessments (For details, please refer Appendix for Training Manual). The accuracy and appropriateness of the study methods will be tested on the role models. Intra and inter observer reliability will be assessed by comparing repeat measurements performed on the same individual at different times by the same observer, and the same individual at the same time by the team of observers.

### Pre-testing

The objectives of pre-testing are to be sure that the:

- Interviewer are comfortable with the questionnaires
- Patients understand the questionnaire
- Questionnaire is appropriate according to the objectives of the study
- All the BP monitors works properly
- Data-entry program is compatible with the questionnaire

On completion of training, research team will go in the field to pre-test questionnaire. It will be done in about 10% of the sample size. The subjects, who would be selected for pre-testing, would not be the part of pilot study.

The entire process of pre-testing will be thoroughly observed by a group of observers including research manager for quality assurance. At the end of pre-testing all shortcomings including need of retraining of interviewers in some areas, revision of questionnaire and management problems in logistics will be addressed appropriately.

### Data Collection

The field team will pay home visits for inviting all enrolled subjects to participate in the study. Informed consent will be obtained. Reasons for not wishing to participate will be recorded. The subjects whose houses will be locked or those who will be out of city will be visited up to three times by the field team and thereafter, they will be labelled as not located. The data collection will be started from April 01, 2012 and anticipated to be completed by March 30, 2013. Following tools will be used for data collection:

*Questionnaires:*

The questionnaires are designed to be interviewer administered, prepared in English, All questionnaires will be translated in to Urdu and back translated and verified before starting the study. Following questionnaires will be used in the study

Form A (For individuals 40 years and above)

Form B (For individuals 15 – 39 years)

Form C (For children 11 – 14 years)

Cardiovascular Morbidity / Mortality questionnaire (For high risk group)

Verbal Autopsy (For high risk group)

Blood Pressure Measurements:

The precise measurement of BP is essential for valid comparisons to be drawn. Therefore a strict routine for BP measurement will be followed. In this study calibrated automated “OMRON Blood Pressure Monitors will be used for measuring BP of the study subjects. Blood pressure will be measured in the sitting position from the right arm after 5 minutes of rest using an appropriate sized cuff. The selection of appropriate size of cuff will based on the mid arm circumference (mid-point between the shoulder and elbow). It will be measured by measuring tape. There are three types of cuffs available for different mid arm circumferences:

Small cuffs: for individuals with arm circumference 17 – 22 cm

Large cuff: for individuals with arm circumference 22 – 32 cm

Extra large cuff: for individuals with arm circumference 32 – 42 cm

Three consecutive readings with an interval of about 05 minutes will be taken on the day prior to initiation of dietary intervention and the mean of the final two blood pressure readings will be used in the analysis as baseline blood pressure.

For the outcome assessment during study period, three consecutive readings of blood pressure with an interval of 05 minutes will be taken at each visit, and the mean of the final two readings on the last day of each intervention week will be used in the analysis.

Anthropometric Measurements

Height will be measured in centimetres with a height scale placed vertically to the hard flat wall with base at the floor level. Subjects will be advised to stand bare footed, looking straight with their back to the height scale, feet together and with their arms hanging loosely by their side.

Body weight will be measured in kilograms (0.1 kg accuracy) by using calibrated “Tanita Solar Powered Digital Scale 1631”. The scale will be placed on to a flat, hard surface directly exposed to room light. Subjects will be asked to remove their shoes, heavy outer garments like Jacket, coat, etc. and stand on the scale with evenly distributed weight on both feet.

Echocardiography:

The echocardiography of all hypertensive patients will be performed at field site by a trained technologist.

## Data Management

The Data will be collected in the 2^nd^ half of the day and will be supervised in the field by spot checking of the filled questionnaires by supervisor. After data collection, the questionnaires will be reviewed next day in the morning by the data collectors and self-edited. If there are any discrepancies found then the questionnaire will take to the field on next day by the same data collector, and will be corrected. After collection, thorough scrutiny of all the completed questionnaires will be done at the CHS department of Aga Khan University by field supervisor and data editors. Coding of the data will be done by data editor with the preparation of codebook after discussion with data manager and research coordinator. The data manager or research coordinator would randomly check filled questionnaires before entry into the computers to ensure the data quality. Data will be double entered in EpiData 3.1 software by two different data entry operators and then be validated and corrected for any disparity by using Microsoft FoxPro/Access. After removing data entry discrepancies, the data will be exported to SPSS and SAS for analysis.

Finally, 10% of the questionnaires would be checked randomly to look for any error in data entry and for calculating the data entry error. If the error rate <0.3% then data would be considered clean. After random checking the frequencies will be run on data for logical error checking. If there are any logical errors found then the study subject will be contacted via phone or field visit to remove those errors. Thereafter, the clean data will be ready for further analysis.

# PLAN OF ANALYSIS:

- SAS version 9.13 will be used for analyses. A p value of <0.05 will be considered statistically significant. All analyses will account for clustering at the level of census by household, as this was also the unit of randomization.[^62^](#_ENREF_62)

## Primary & Secondary Outcomes:

For the main intention to treat analysis, the mean estimated value in each cluster for those with missing values at the final visit will be computed for all individuals with available measurements, and assigned to those with missing last follow-up measurements for individuals by cluster. This method of imputation of missing values has been shown to yield valid inferences and is recommended for large community intervention trials with small size clusters.[^63^](#_ENREF_63),[^64^](#_ENREF_64) The primary and secondary outcomes will be compared between randomised clusters using a nested mixed factorial design analysis of variance. Due to previously detected significant interaction between treatment assignments on BP in the high risk group, only four-way analysis will be performed in that group.[^65^](#_ENREF_65) A p value of <0.05 was considered to be statistically significant for the main effects. We will report the means and 95% confidence intervals for the treatment effects, adjusted for sex, age and baseline BP in the models. In addition, models will also be constructed after accounting for the baseline and post-trial measurement of fasting plasma glucose, and lipids (as indicated). In the model for CVD morbidity and mortality, albuminuria, LVMI, and E/E’ ratio we will also adjust for change in systolic BP from baseline to follow-up. The marginal adjusted probabilities will be used to calculate the risk ratios, and their 95% CI will be computed by the bootstrapping method.[^66^](#_ENREF_66)

## Ancillary Analysis:

a) For the quality of life study, the mean overall HRQoL score of the high risk cohort will be computed, and compared across randomized groups. Separate multivariable models will be built for the outcome of mean HRQoL score. Linear regression analysis will be performed to determine the association of a) systolic BP at baseline, b) systolic BP at follow-up; c) change in systolic BP from baseline to follow-up with and without adjusting for current use of antihypertensive medications; and b) use of antihypertensive medications. The other candidate predictors will include age, sex, level of education, and socioeconomic status.

b) For the GP survey, the means and proportions of the responses will be compared, as appropriate, for GPs in the clusters randomized to trained GP versus usual care, accounting for clustering by census. The analysis will be done per intention-to-treat as well as per-protocol (restricted to trained GP in relevant clusters).

c) For the assessment of the relationship of BP variability with LV mass and CV morbidity and mortality: BP was measured at baseline and then average of 6-monthly intervals on all high risk trial participants. All available BP readings measured at baseline and 6 monthly intervals during 2 years of intervention on high risk group will be used. We will define four metrics of BP variability based on crude systolic and diastolic BP data: 1) the standard deviation, 2) the coefficient of variation (standard deviation divided by mean BP), 3) variation independent of mean, proportional to SD/mean X, where X will be estimated for each subject by fitting a mixed-effects linear regression model of time on observed BP, which will account for greater fluctuations with higher mean BP.[^50^](#_ENREF_50),[^67^](#_ENREF_67)

We will build multivariable models for the outcomes of LVMI, E/E’ ratio and composite of CVD morbid events and all-cause mortality during 6 years. Candidate BP predictors will include BP variability, absolute baseline BP, mean of all follow-up readings, peak systolic BP achieved during any one of the visits, and episodic stage 2 systolic hypertension (systolic BP >= 160 mm Hg) during these visits. Other predictors will include age, sex, baseline BMI, tobacco use, fasting plasma glucose, and fasting blood lipids. Stepwise forward selection will be used in logistic regression time series analysis with random effects.[^68^](#_ENREF_68) All analyses will account for treatment assignment and clustering.

## Ethical Considerations

Ethical approval will be sought from the Ethics Review Committee at the Aga Khan University. Informed consent will be obtained from each adult and, where children are involved, assent from the child and consent from parents prior to enrolment.

All participants enrolled in the main COBRA trial will be included in the study for follow-up. However, the individuals (children or individuals aged 15 – 39 years) not enrolled in the main study, pregnant women, bed ridden and/or mentally retarded study subjects unable to consent will be excluded.

Since, this is a follow-up study on the participants enrolled earlier in the main study, there are no major ethical issues in this follow-up study. However, we will make sure that following normal ethical issues should be dealt ethically:

- Written consent and assent will be obtained from adults and children participants respectively.
- It would be made clear to each and every participant that he/she is free to decide whether or not to participate in this follow-up study.
- The participants will be briefed about the nature of the study, the interview technique, the potential nature of use of the information collected, type of medical examination performed and the type of investigations taken.
- He/she will be free to withdraw from this study at any time without giving any reason for withdrawing.
- The expenses for the all laboratory tests performed during this follow-up will be covered by the study.
- Study participants unwilling for laboratory tests will not be forced for blood / urine sampling.
- The participants with cardiovascular events during the study will be assessed and advised by study cardiologist /neurologists for management of complication and prevention of future events.
- If high BP with headache or chest pain or shortness of breath or dizziness or blurred vision or any other active disease is diagnosed during assessment, the participant will be referred to the hospital for appropriate management. A referral letter and copy of laboratory tests will be provided.

*Confidentiality of the subjects:*

- All the collected identifiable information and reports will be kept confidential in a secure locked space.
- All filled questionnaires would be kept in secure space with restricted access exclusively to key research team members (PI, coordinator, and data manager).
- Electronic copies of the complete data will be stored in password protected data files.
- Only anonymized and de-linked data will be shared with research team members for analysis.

*Risk and benefits to the study subjects:*

No adverse effects / risks are expected as this is an observational follow-up without any intervention. The study subjects will have the same benefits as those of main study, i.e.

- All participants during follow-up will have their free physical assessment and advice for follow-up with appropriate referral to a family health care provider.
- The subjects enrolled during the follow-up would be evaluated for hypertension, coronary artery disease diabetes, dyslipidemia and albuminuria through blood and urine tests.
- The cost of all blood and urine tests, and echocardiography performed during this follow-up will be covered by the study.
- The reports will be communicated to them along with appropriate advice.
- The participants with positive cardiovascular events will have their assessment by cardiologist / neurologist free of charge. Moreover, they will have a free pick and drop facility.

# TIME LINE:

Protocol development, and ethics approval: 3 months, implementation and data collection 12 months; analysis; 9 months (aims 1 & 2: 4 months; aims 3 & 4: 4 months).

**
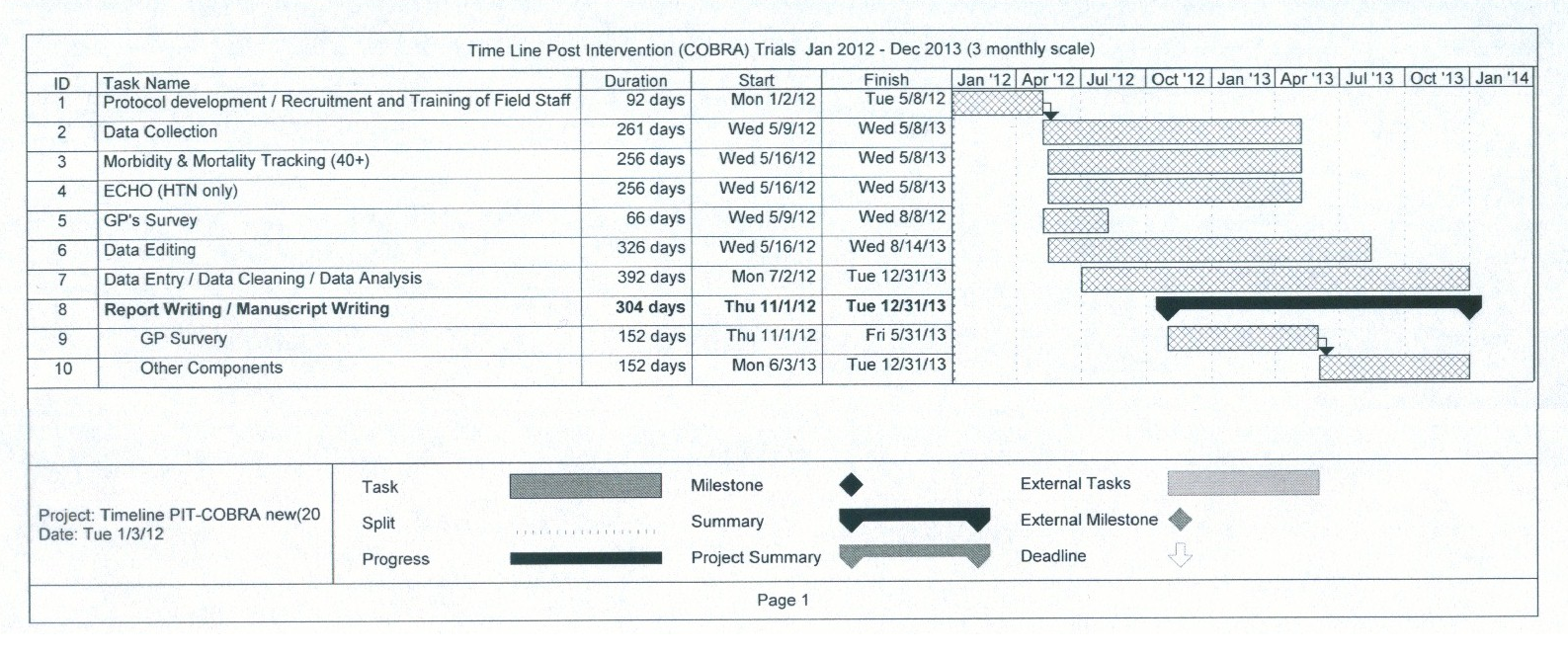
**

# REFERENCES:

# 
